# Supplementary material for: ROS Signaling Mediates Directional Cell Elongation and Somatic Cell Fusion in the Red Alga Griffithsia monilis
Source: Cells. 2022 Jul 5;11(13):2124. doi: 10.3390/cells11132124 (PMC9266221; doi:10.3390/cells11132124)
Supplement: Supplementary file 1 [file cells-11-02124-s001.zip › Supplementary Figures S1-S4 and Table S1.pdf]

Supplementary Figure S1

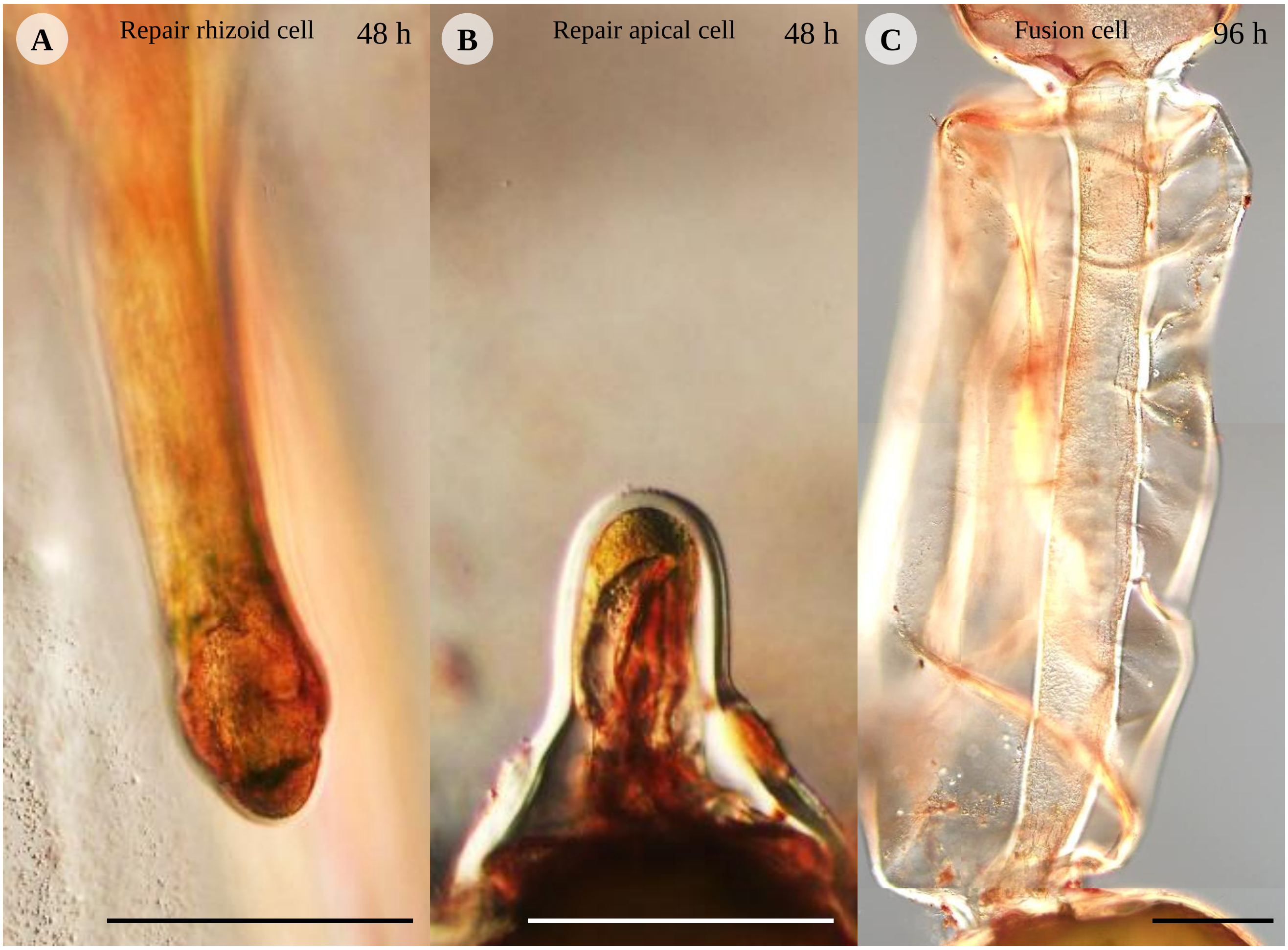

Supplementary Figure S2

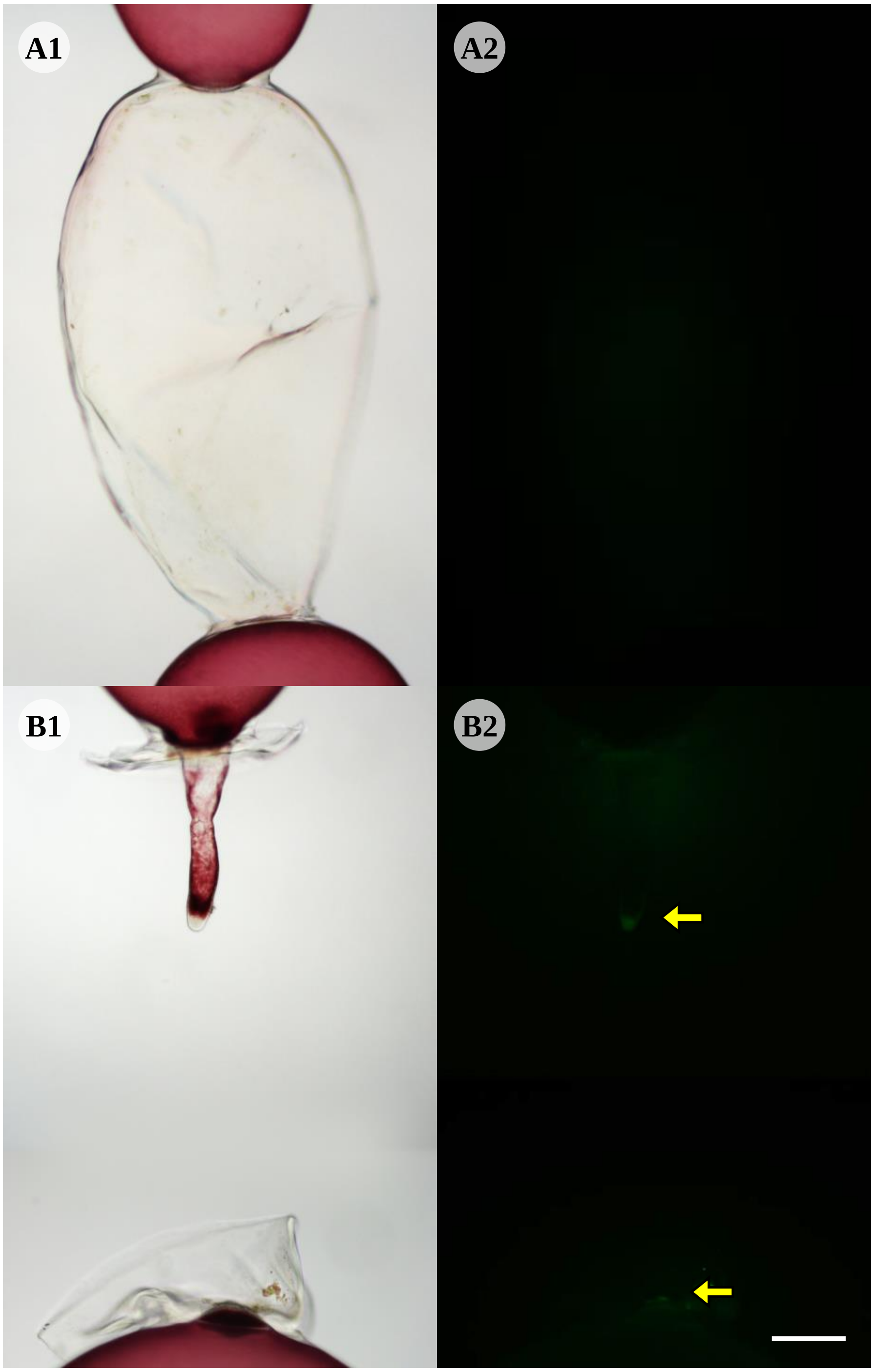

Supplementary Figure S3

FAD-binding domain

NADPH-binding domain

5′ or 3′-RACE PCR primer (GSP) binding site

Start or stop codons

A

GmRBOH1

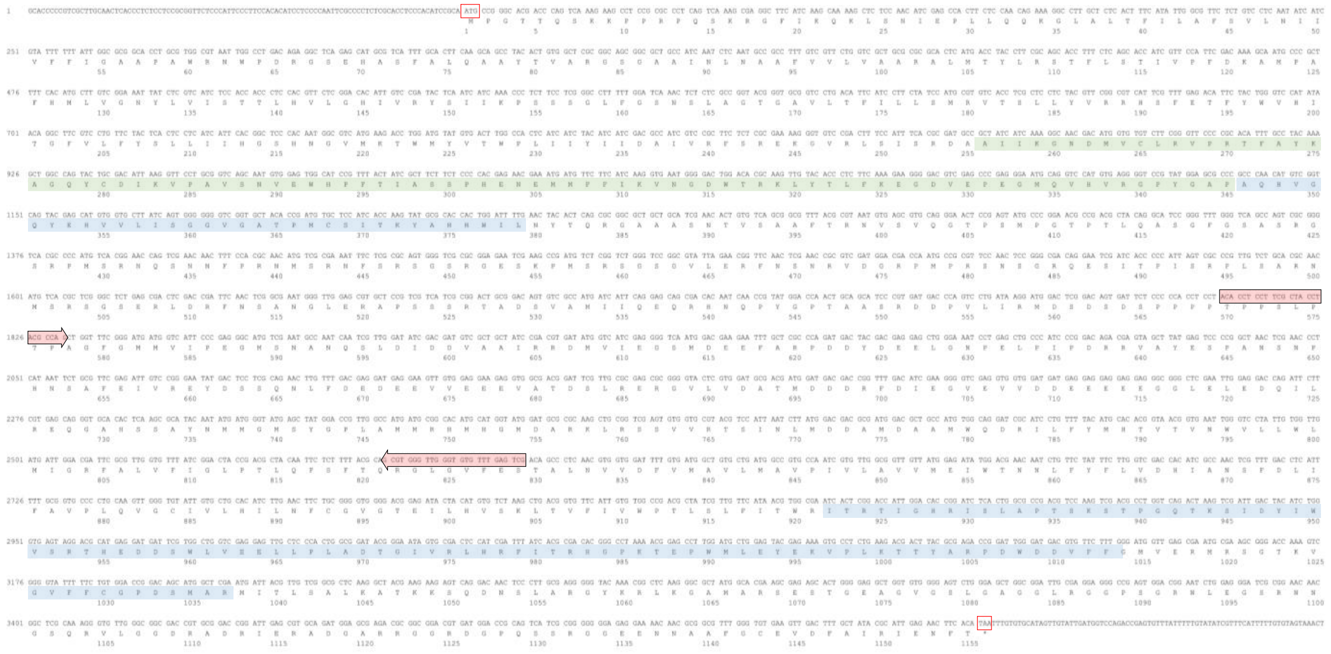

GmRBOH2

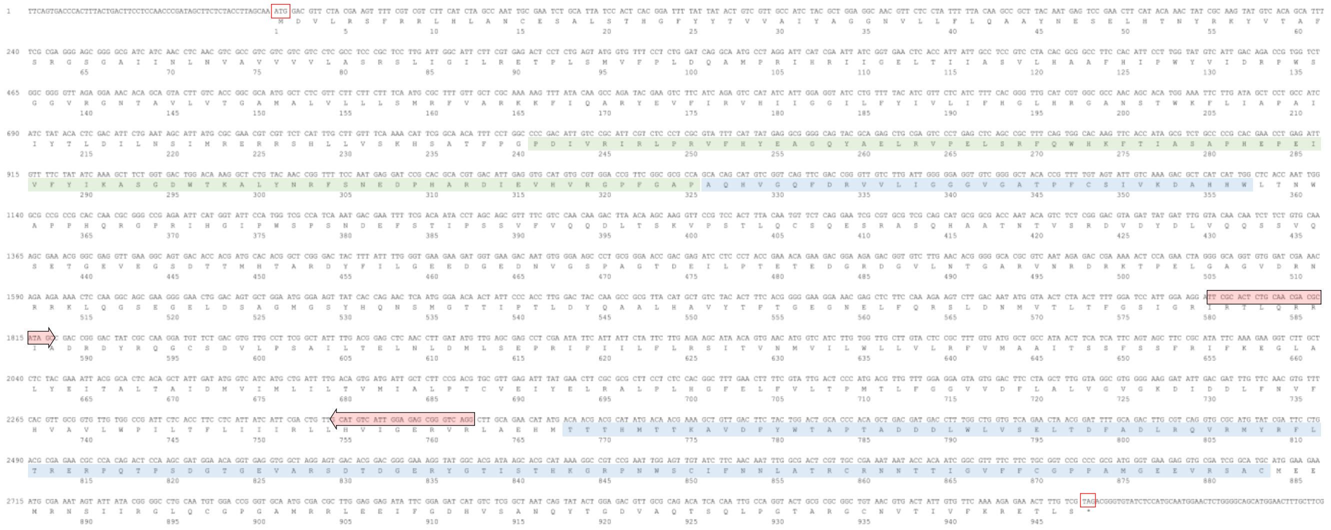

Supplementary Figure S3

GmRBOH3

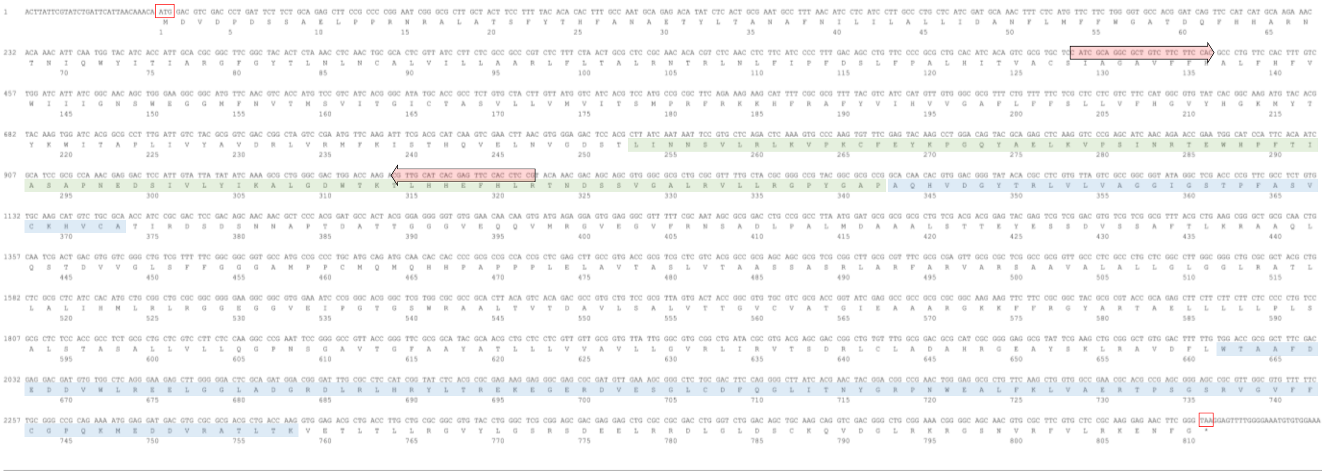

GmRBOH4

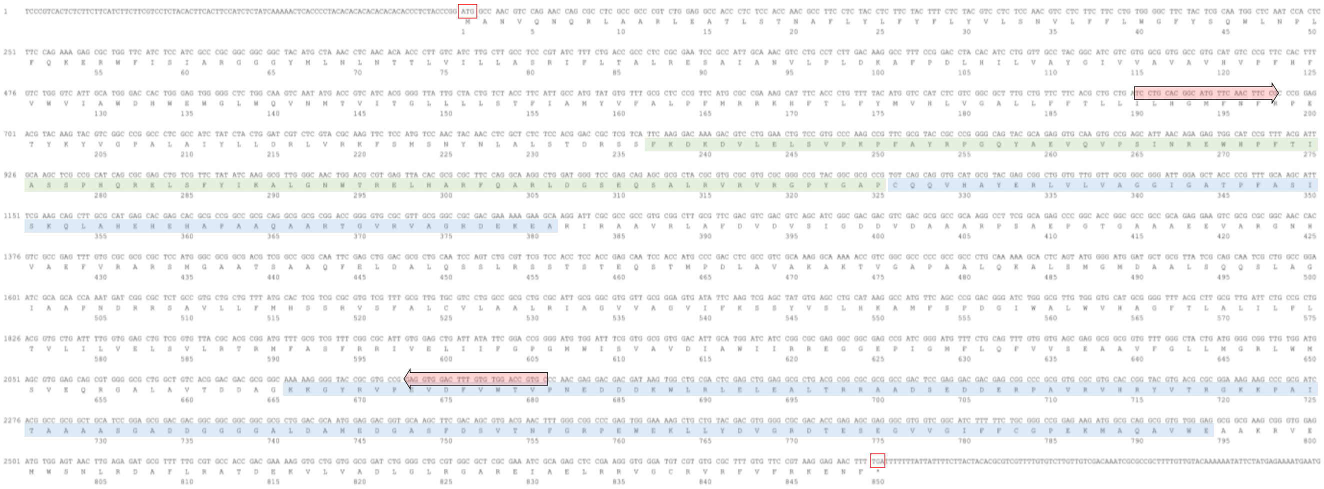

Supplementary Figure S3

B

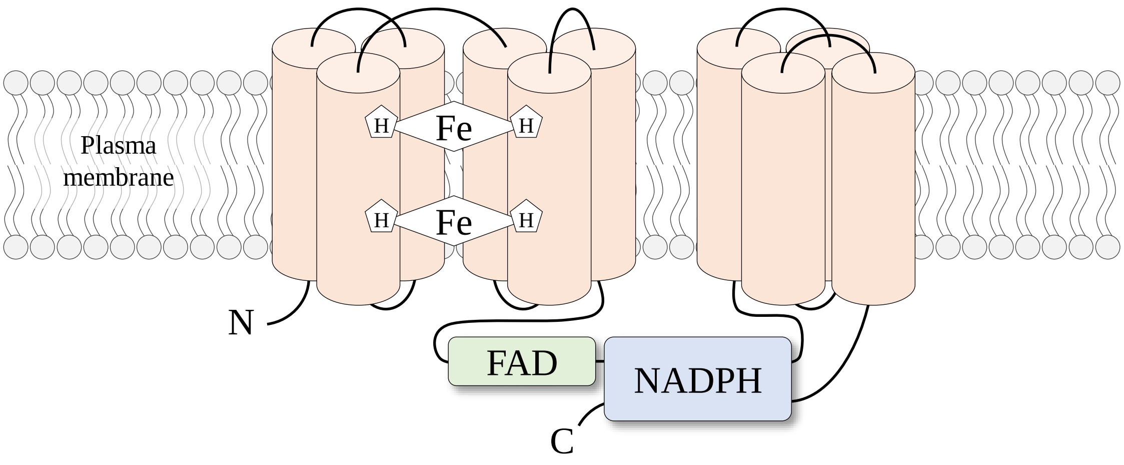

EF, EF hand; FAD, FAD-binding domain; H, Heme motif; NADPH, NADPH-binding domain; TM, transmembrane domain

Supplementary Figure S4

Ascorbic acid

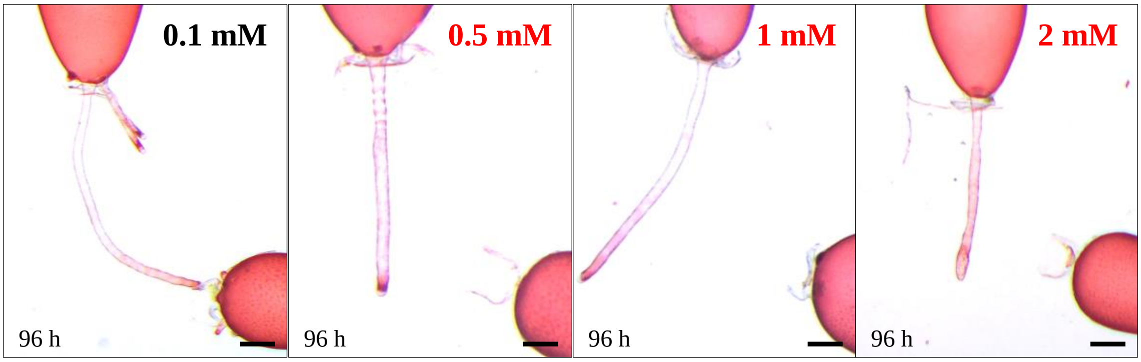

	0.1 mM	0.5 mM	1 mM	2 mM
Ascorbic acid	-	+	+	+

DPI

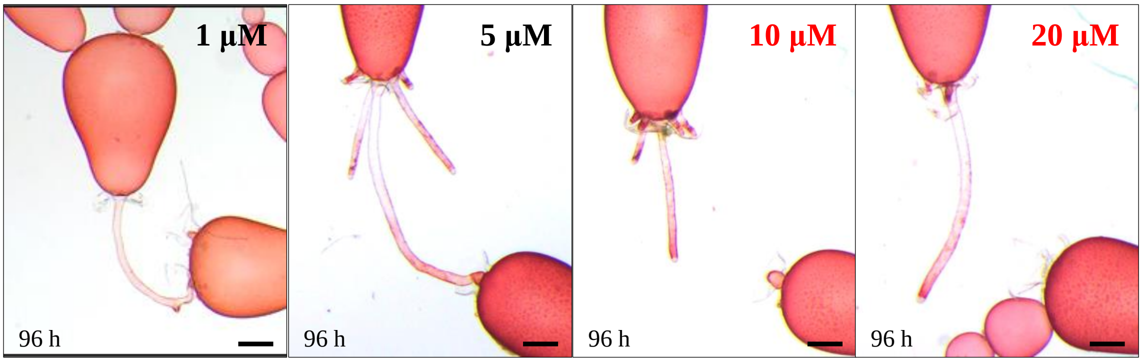

	1 µM	5 µM	10 µM	20 µM
DPI	-	-	+	+

Caffeine

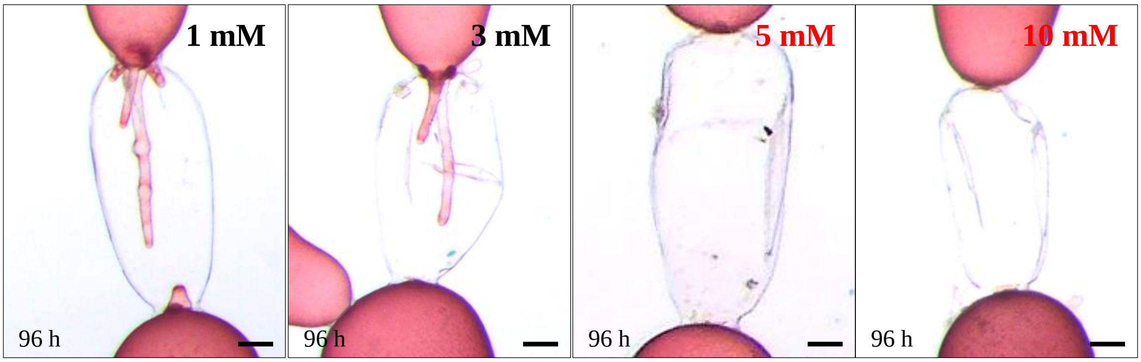

	1 mM	3 mM	5 mM	10 mM
Caffeine	-	-	+	+

H_2O_2

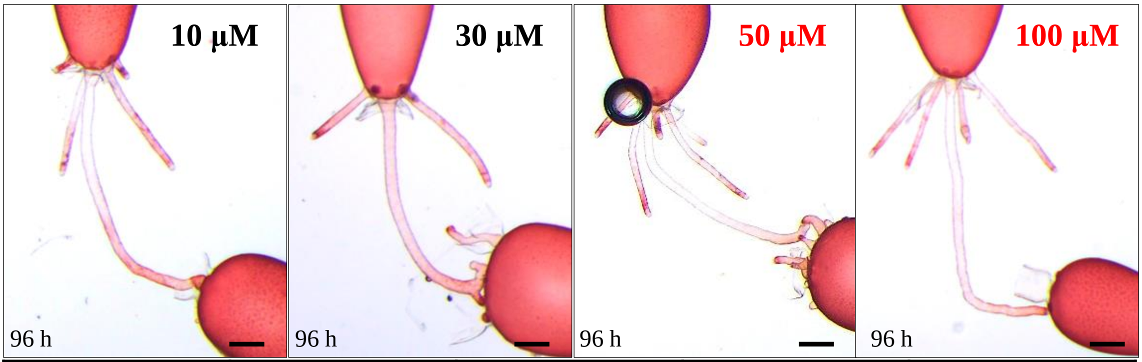

	10 µM	30 µM	50 µM	100 µM
H_2O_2	+	+	++	++

Supplementary Table S1

Name	Sense primer	Antisense primer
GmRBOH1-5’-RACE-GSP		GATTACGCCAAGCTTCGACTCAAACACACCCAACCCACGC
GmRBOH1-3'-RACE-GSP	GATTACGCCAAGCTTACACCTCCTTCGCTACCTACGCCAG	
GmRBOH2-5'-RACE-GSP		GATTACGCCAAGCTTCCTGACCCGCTCTCCAATGACATGC
GmRBOH2-3'-RACE-GSP	GATTACGCCAAGCTTTTCGCACTCTGCAACGACGCATAGC	
GmRBOH3-5'-RACE-GSP		GATTACGCCAAGCTTCGGAGGTGGAACTCGTGATGCAACG
GmRBOH3-3'-RACE-GSP	GATTACGCCAAGCTTCATCGCAGGCGCTGTCTTCTTCCAC	
GmRBOH4-5'-RACE-GSP		GATTACGCCAAGCTTGCACGGTCCACACAAAGTCCACCTC
GmRBOH4-3'-RACE-GSP	GATTACGCCAAGCTTTCCTGCACGGCATGTTCAACTTCCG	
GmEF1A-qPCR	AGGTGGAGGATCCGGACTAC	CCCTCGCATTGCTTGAATCC
GmRBOH1-qPCR	GATTGAGCGTGCAGATGGAG	GTCAACTTCACACCCAAACG
GmRBOH2-qPCR	GAAATTACGGCACTCACAGC	GCGCGCGAAGTTCATAAATC
GmRBOH3-qPCR	CAGATGGACGGGATTTGCG	GTGATAAGCCCCTGGAAGTC
GmRBOH4-qPCR	GTTGTGGATGAGCGTGGAG	CACGGTCCACACAAAGTCC
